# Assessing soil quality of Italian Western Alps protected areas by QBS-ar: impact of management and habitat type on soil microarthropods

**DOI:** 10.1007/s10661-023-11880-9

**Published:** 2023-10-10

**Authors:** Tommaso Fusco, Lorenzo Fortini, Francesca Casale, Carlo Jacomini, Andrea Di Giulio

**Affiliations:** 1 Department of Science, University Roma Tre, Viale G. Marconi, 446 –, 00146 Rome, Italy; 2 Biology Area, Soil and Land Ecology Laboratory, Italian Environmental Protection and Research Institute (ISPRA), National Centre for the Italian Laboratories Network (CN_LAB), Via V. Brancati, 48 –, 00144 Rome, Italy; 3 NBFC, National Biodiversity Future Center, 90133 Palermo, Italy

**Keywords:** QBS-ar, Microarthropods, Soil monitoring, Forest management, Soil quality, Biological index, Parco Naturale delle Alpi Marittime

## Abstract

Soil fauna has a crucial importance for the functioning of ecosystems and their conservation. Soil biota has a role in soil formation and distribution of organic matter, and groups like microarthropods can be used as indicators to assess soil quality and are often employed in monitoring programs. In the present study, the QBS-ar index, an index based on the presence/absence of microarthropod groups, was used to assess the level of soil quality in nine different sampling sites in the “Parco Naturale delle Alpi Marittime” and in the “Parco Naturale del Marguareis” (Cuneo, Piedmont). Forest soils, with different degree of management, and open environments (e.g., grasslands and peatlands) were analyzed comparatively, to investigate whether microarthropod fauna might be influenced by management and habitat type.

The results show QBS-ar values are significantly higher in woodland soils compared to grasslands and peatlands (*p* < 0.05). The latter shows no significant difference between each other, although grasslands show a large range of values (108–214). Forest management does not seem to influence QBS-ar values (183–239), showing stable microarthropod communities both in the managed and unmanaged areas. In addition to this, QBS-ar values do not differ significantly in the different forest coenoses, confirming that woodlands have similar index values (*p* = 0.7).

This study confirms that QBS-ar values in natural areas can vary depending on the environment. It is therefore important to consider clustering habitat types before assessing quality classes for QBS-ar values. Finally, sustainable forest management in the study area does not seem to affect significantly soil microarthropod presence in woodland sites.

## Introduction

Several efforts invested in soil monitoring in Europe did not bring to a comprehensive and updated body of knowledge for identifying healthy soils and those that are degraded and require protection (EEA, 2022).

Soil quality can be defined as “the capacity of the soil to promote the growth of plants, protect watersheds by regulating the infiltration and partitioning of precipitation, and prevent water and air pollution by buffering potential pollutants such as agricultural chemicals, organic wastes, and industrial chemicals” (National Research Council, 1993). Important component of soil quality assessment is the identification of a set of sensitive soil attributes that reflect the capacity of a soil to function and can be used as indicators of soil quality (Bünemann et al., 2018).

Soil fauna represents a part of biodiversity that is far from being fully studied. However, it has a crucial importance for the functioning of ecosystems and their conservation, as it plays a substantial role in plant growth and primary production (Maharning et al., 2009). Soil communities are important in the soil formation process because they influence the distribution of organic matter and decomposition rates (García-Palacios et al., 2013; González & Seastedt, 2001; Njoroge et al., 2022). Many edaphic organisms are detritivores and decomposers and act on organic remains keeping the soil fertile and nutrient rich (Menta, 2012). Moreover, the limited vagility of soil mesofauna provides for an effective indication of the effects of stress factors on the conditions of the soil cores, and the sensitivity to environmental stress such as chemical, physical, and biological pollution has been tested on a bulk of situations (Ojala & Huhta, 2001).

Therefore, the richness and diversity of animal taxa and the complexity of the edaphic communities in a given area can be indicative of the level of maturity of the ecological community. The process of succession results in increased structure, stability, and energy in the ecosystems, which facilitate the development of high trophic levels (Menta, 2012).

Because many groups belonging to the meso- and macrofauna are particularly sensitive to environmental stresses, in particular soil microarthropods, they can be used as indicators to assess soil quality and are often employed in monitoring programs. For this reason, different edaphic groups have been used in the last 20 years to create different types of indices (QBS-ar, QBS-c, IBSQ, QBS-e, QBS-BF, FAI, etc.) based on abundance, presence or absence, and diversity (Parisi, 2001; Parisi et al., 2005; Parisi & Menta, 2008; Nuria et al., 2011; Santorufo et al., 2012; Yan et al., 2012; Paoletti et al., 2013; D'Avino et al., 2022).

In the present study, we applied QBS-ar index, or index of soil biological quality, proposed in 2001 to assess the level of soil quality using the presence/absence of edaphic microarthropods as a parameter (Parisi, 2001; Parisi et al., 2005). QBS-ar is based on the assumption that the single presence is sufficient to represent the soil adaptability of that group. The main advantage of this index is that, unlike indices that use a single taxon as biological indicator, and require advanced taxonomic knowledge, it does not require identification at the species level, but only at the order or class level. This makes it possible to simplify and speed up the process of assigning a soil quality score. In addition, compared with other indices (Aoki, 1977; Bachelier, 1986), QBS-ar does not require counting individuals in the sample, but it is sufficient to know which biological forms are present. Nuria et al. (2011) proposed an integrated approach called IBQS (Synthetic Index of Biological Quality of Soil) that assesses soil quality by considering macro-invertebrate communities, which are directly involved in ecosystem services. However, Menta and Remelli (2020) showed that the IBQS index was affected by the intensity level of management practices. Another index developed in the last few years is the IBS-bf (Soil Biodiversity Index) (Caoduro et al., 2014). Both protocols showed the same trends. The highest values were recorded in natural areas, intermediate values in organic farming, and lower values in integrated production farms (Menta et al., 2015).

Assessing fluctuations in soil quality is critical to assess the health of an ecosystem (Schoenholtz et al., 2000), and the QBS-ar index is a rapid and inexpensive approach to characterizing edaphic communities and assessing soil quality.

The recent use of the QBS-ar in various countries such as Chile, Nepal, India, Mexico, and other European countries besides Italy (Çakır et al., 2023; Galli et al., 2021; Hernández-Tirado et al., 2022; Menta et al., 2018; Shrestha & Budha, 2022; Szigeti et al., 2022) shows that this index is being adopted worldwide.

The sampling was carried out as part of the COBIODIV project, which aims to acquire data on the unknown Alpine biodiversity between the Italian and French borders (Schatz et al., 2021).

In this study, for the first time within the Maritime Alps (Piedmont, Italy), we analyzed comparatively soils from both forests (with different composition and type of management) and open environments (e.g., grasslands, pastures, and peatlands) using the QBS-ar index. Additionally, the comparison of QBS-ar values between woodlands, grasslands, and peatlands allows to discuss improvements to the QBS-ar elaboration, which might get a better sense if possibly rescaling the index quality classes when values are clustered and weighted based on plant coenoses or habitat types.

## Materials and methods

### Study area

**Fig. 1:**
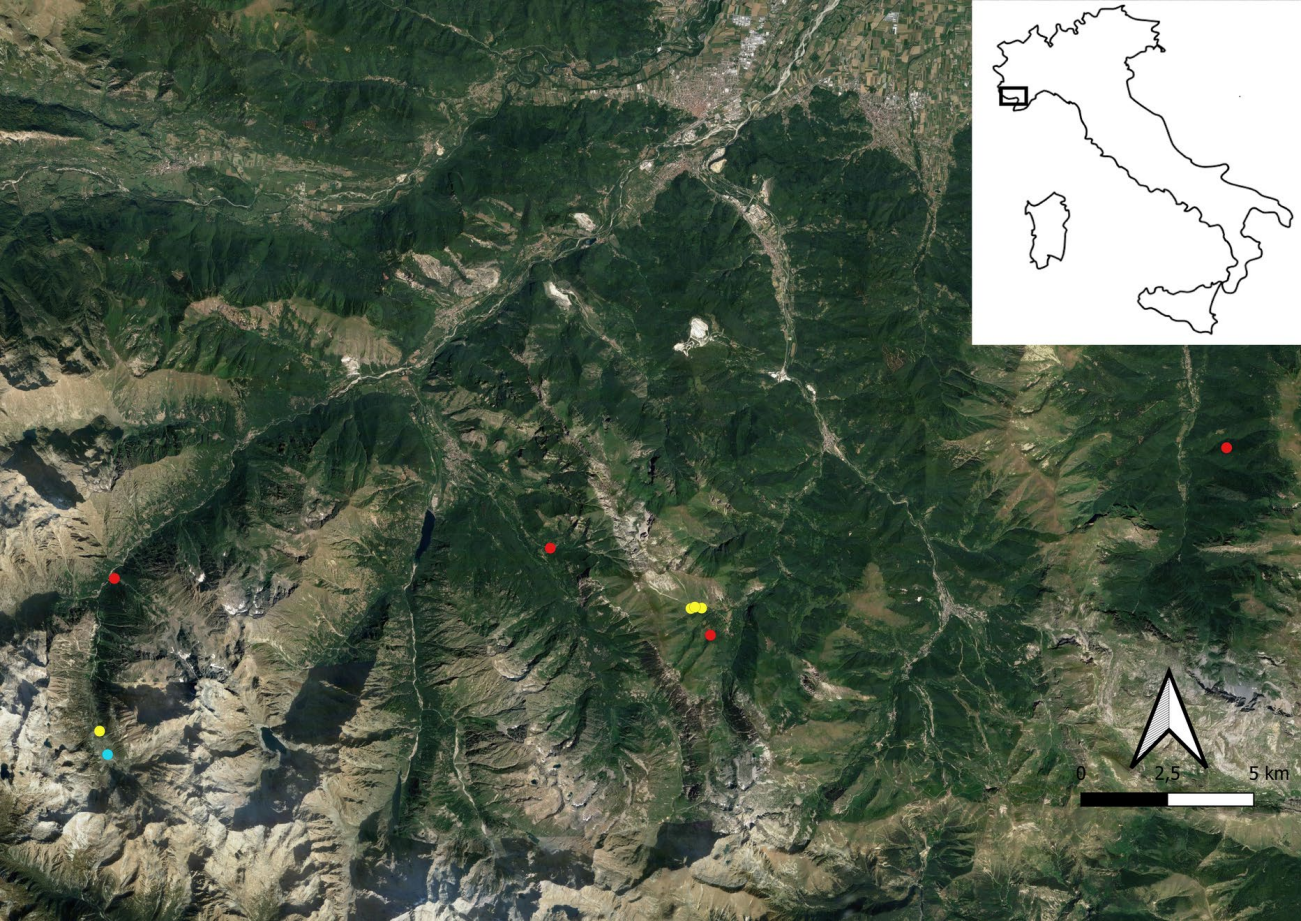
Study area. Red dots = woodlands; yellow dots = grasslands; blue dot = peatland

For this study, nine sampling sites were chosen for this research, eight in the “Parco Naturale delle Alpi Marittime” and one in the close-by “Parco Naturale del Marguareis,” all in the province of Cuneo (Piedmont, Italy). Sampling campaigns took place in July 2020 and June 2021 and three different types of habitats were chosen: 4 woodland sites, 4 grassland sites, and 1 peatland site (Fig. 1).

The woodland sites differed in compositional type: one ash forest in locality Tetti Prer (ASH), one fir forest in locality “Certosa di Pesio” (FIR), and two beech forests, respectively, in locality “Terme di Valdieri” (BEV), and “Palanfrè” (BEP). Besides vegetation type, the forest sites differ in forest management (coppice with log removal—managed vs. undisturbed forest—unmanaged). Further details on sampling sites are reported in Table 1.

**Table 1:** Characteristics of sampling sites

s	Locality	Date	Latitude	Longitude	Altitude	Tree cover	Herbaceous cover	Rock outcrop	Inclination	Dominant plant species
ASH	Tetti Prer	17-VII-2020	N44.21648°	E7.43319°	1119–1130 m a.s.l.	40–80%	80%	Low	0–5%	*Fraxinus excelsior*
FIR	Certosa di Pesio	18-VII-2020	N44.24245°	E7.67773°	1128–1155 m a.s.l.	70%	30%	Medium	30–50%	*Abies alba*
BEP	Palanfrè	19-VII-2020	N44.19395°	E7.49112°	1572–1631 m a.s.l.	80–90%	5%	Low	50–60%	*Fagus sylvatica*
BEV	Terme di Valdieri	19-VII-2020	N44.20863°	E7.27559°	1411–1454 m a.s.l.	80–90%	5%	High	20%	*Fagus sylvatica*
PRATO	Palanfrè	19-VII-2020	N44.20097°	E7.48792°	1495–1498 m a.s.l.	<5%	100%	Low	20%	Herbaceous
PAS	Palanfrè	30-VI-2021	N44.20086°	E7.48405°	1584–1609 m a.s.l.	0%	100%	Low	10%	herbaceous
FdR	Palanfrè	30-VI-2021	N44.20122°	E7.48549°	1531–1556 m a.s.l.	0%	100%	Low	40–50%	herbaceous
ALP	Gias della Casa	29-VI-2021	N44.16910°	E7.27025°	1730–1738 m a.s.l.	0%	100%	Low	10%	herbaceous
TOR	Pian della Casa	29-VI-2021	N44.16301°	E7.27313°	1761–1768 m a.s.l.	0%	100%	Low	0–5%	herbaceous

### QBS-ar protocol

Sampling and extraction of soil microarthropods followed the methodology recommended for the application of the QBS-ar protocol (Parisi et al., 2005) which can be divided in 5 phases: (1) sampling, (2) microarthropod extraction, (3) preservation of the collected specimens, (4) determination of biological forms, and (5) calculation of the QBS-ar index.

Before extracting the sample, the herbaceous cover and a part of the litter were removed using garden shovels and scissors. In each site, three 10 × 10 × 10 cm soil cores were removed using a soil corer and immediately placed in a plastic bag that was then sealed, leaving an air reserve to allow the micro-arthropods to survive until the moment of extraction.

Additionally, in order to make a comparison between QBS-ar values in managed and unmanaged areas in woodland sites, a total of six soil cores were sampled (3 from managed and 3 from unmanaged areas).

For each sampling site, different data were recorded. Tree, shrub, herbaceous, and litter cover were recorded in the field as a percentage, slope was measured in degrees, and rockiness was estimated using three levels (low, medium, high) (Table 1).

Berlese-Tüllgren funnels were used for microarthropod extraction. Each sampled soil replicate was placed on a steel sieve with 30 cm diameter and 2-mm mesh. A Falcon tube filled with 70% ethyl alcohol and about 5 ml of glycerin was placed under the funnel, to prevent excessive evaporation of alcohol during extraction. Halogen lights (60 W) were placed 40 cm above the samples and were turned on the day after installation of the extractors to stabilize the experimental conditions. The extraction of the microarthropods lasted 12 days.

The organisms were subsequently sorted under a stereo microscope (OLYMPUS SZX16) and using identification keys. In this phase, soil organisms are also separated into biological forms according to their morphological adaptation to soil environment; each of these forms is associated with a score called EMI (eco-morphological index), which ranges from 1 to 20 in proportion to the degree of soil adaptation. When more biological forms are present for the same group of organisms, the higher EMI score is taken into consideration (Parisi et al., 2005). The QBS-ar index value of each replicate is obtained from the sum of the EMI of all collected groups. In addition to this, QBS values from the three replicates of each sampling plot were reckoned as one single QBS-ar value (Parisi, 2001; Parisi et al., 2005), assessing the highest value to all biological forms present in the replicates, even if they might be present in only one of them.

### Statistical analysis

To test if there is a statistically significant difference between QBS-ar values in areas where the forest is managed and those not managed, a multi-way ANOVA test was performed.

The QBS-ar index values in different habitats (woodland, grassland, peatland) were analyzed using an ANOVA test to study the statistical differences between them. Tukey’s honestly significant difference HSD test was carried out (post hoc test) to comprehend how specific group means differ.

Before carrying out the tests, the basic assumptions were tested performing a Shapiro-Wilk test, to evaluate the normality of distribution and an *F* test to control homoscedasticity. The data were normally distributed (*p* > 0.05), and the variances were equal (*p* > 0.05) in all cases.

All tests were performed using RStudio version 1.3.1093 (R Development Core Team, 2021).

## Results

The results in Table 2 show that the highest QBS-ar values for the replicates correspond to woodland habitats and vary between 224 and 107 with a mean of 169.87 and the highest value registered in the ash wood forest. The lowest values were obtained in the peatland site TOR with a mean of 84, while the grassland shows a range of values between 160 and 78 and a mean of 114.75.

**Table 2:** QBS-ar values for each site and replicate

Site	Management	QBS-ar	Replicate	Composition
ASH	Unmanaged	107	*R*1	Woodland
ASH	Unmanaged	175	*R*2	Woodland
ASH	Unmanaged	224	*R*3	Woodland
ASH_UM_MAX	**Unmanaged**	**238**	**All**	**Woodland**
ASH	Managed	179	*R*1	Woodland
ASH	Managed	199	*R*2	Woodland
ASH	Managed	199	*R*3	Woodland
ASH_M_MAX	**Managed**	**220**	**All**	**Woodland**
BEV	Unmanaged	199	*R*1	Woodland
BEV	Unmanaged	191	*R*2	Woodland
BEV	Unmanaged	218	*R*3	Woodland
BEV_UM_MAX	**Unmanaged**	**239**	**All**	**Woodland**
BEV	Managed	112	*R*1	Woodland
BEV	Managed	107	*R*2	Woodland
BEV	Managed	147	*R*3	Woodland
BEV_M_MAX	**Managed**	**183**	**All**	**Woodland**
BEP	Unmanaged	192	*R*1	Woodland
BEP	Unmanaged	127	*R*2	Woodland
BEP	Unmanaged	156	*R*3	Woodland
BEP_UM_MAX	**Unmanaged**	**194**	**All**	**Woodland**
BEP	Managed	187	*R*1	Woodland
BEP	Managed	145	*R*2	Woodland
BEP	Managed	206	*R*3	Woodland
BEP_M_MAX	**Managed**	**211**	**All**	**Woodland**
FIR	Unmanaged	195	*R*1	Woodland
FIR	Unmanaged	152	*R*2	Woodland
FIR	Unmanaged	189	*R*3	Woodland
FIR_UM_MAX	**Unmanaged**	**214**	**All**	**Woodland**
FIR	Managed	127	*R*1	Woodland
FIR	Managed	162	*R*2	Woodland
FIR	Managed	182	*R*3	Woodland
FIR_M_MAX	**Managed**	**193**	**All**	**Woodland**
PRATO	-	89	*R*1	Permanent grassland
PRATO	-	78	*R*2	Permanent grassland
PRATO	-	119	*R*3	Permanent grassland
PRATO_MAX	**-**	**131**	**All**	**Permanent grassland**
PAS	-	160	*R*1	Permanent grassland
PAS	-	128	*R*2	Permanent grassland
PAS	-	120	*R*3	Permanent grassland
PAS_MAX	**-**	**170**	**All**	**Permanent grassland**
FdR	-	150	*R*1	Permanent grassland
FdR	-	123	*R*2	Permanent grassland
FdR	-	131	*R*3	Permanent grassland
FdR_MAX	**-**	**214**	**All**	**Permanent grassland**
ALP	-	94	*R*1	Permanent grassland
ALP	-	87	*R*2	Permanent grassland
ALP	-	98	*R*3	Permanent grassland
ALP_MAX	**-**	**108**	**All**	**Permanent grassland**
TOR	-	83	*R*1	Peatland
TOR	-	92	*R*2	Peatland
TOR	-	77	*R*3	Peatland
TOR_MAX	**-**	**103**	**All**	**Peatland**

Management is indicated only for woodland sites as managed or unmanaged. QBS-ar values are indicated for the single replicates and the maximum value for each site is indicated with “all.” The maximum QBS-ar value is obtained by adding the highest value of EMI for each biological form for each of the three replicates

Maximum QBS-ar values for each site are in bold

Maximum QBS-ar values are higher in forest sites varying from 183 to 239 which correspond to high soil quality. Grasslands have lower and more variable values compared to the latter, ranging from 108 to 214.

**Fig. 2:**
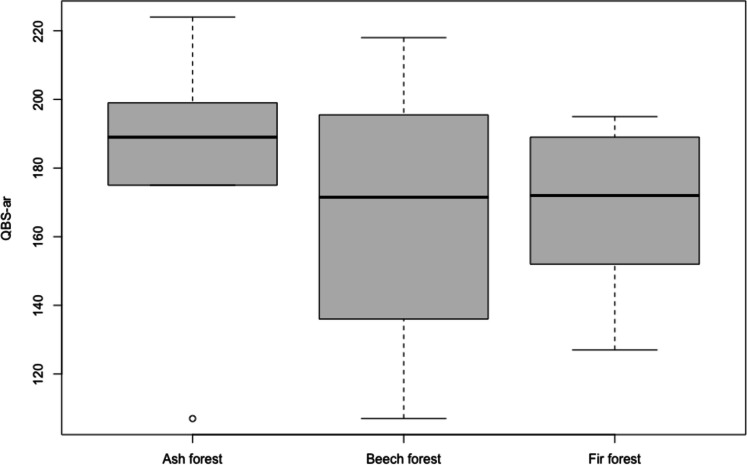
QBS-ar values recorded in different types of forest coenoses

**Fig. 3:**
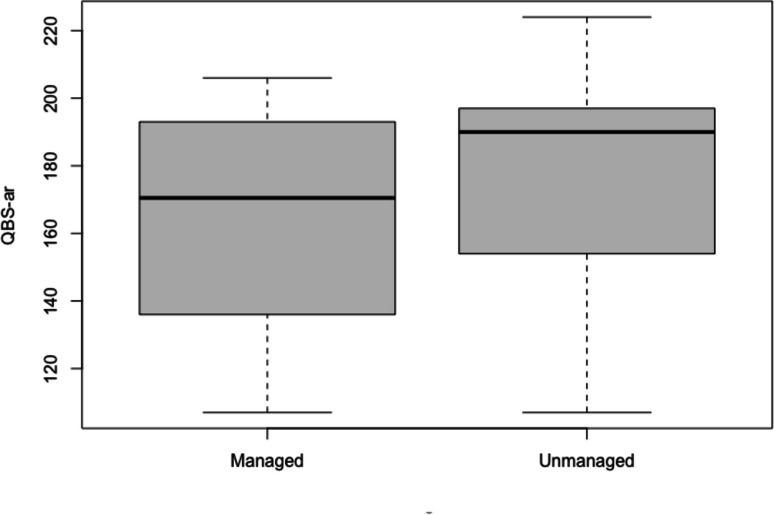
QBS-ar values in managed and unmanaged forests

The obtained results showed that QBS-ar values do not differ significantly in the different forest coenoses, nor between managed and not managed areas and between the interaction of these two factors (Table 3; Figs. 2 and 3).

**Table 3:** Results of ANOVA test

Source	df	Sum sq	Mean sq	F-ratio	p-value
Forest coenoses	2	923	461.7	0.36	0.7
Management	1	1247	1247	0.972	0.3
Forest coenoses/Management	2	2967	1483.7	1.157	0.3
Residuals	18	23,089	1282.7

**Fig. 4:**
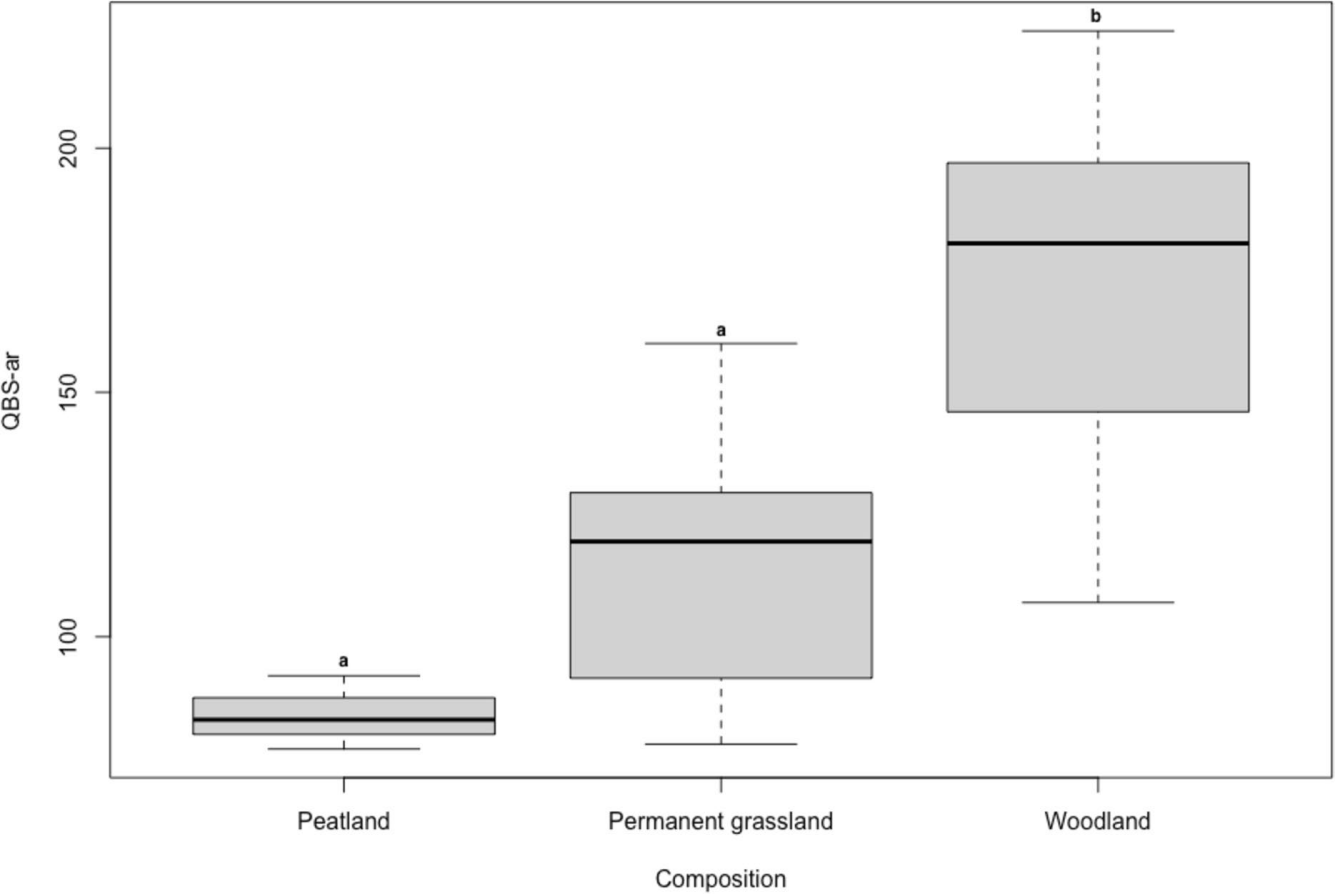
QBS-ar values in different types of habitats

The QBS-ar values are significantly higher in woodland habitats compared to permanent grasslands and the peatland (woodland-grassland *p* < .001, woodland-peatland *p* < .001) but did not differ between the latter (grassland-peatland *p* = 0.29; Fig. 4).

## Discussion

All the soils examined in this study are characterized by high biological activity, with the number of biological forms ranging from 12 to 21, and high QBS-ar values, indicating that the areas investigated have a good state of conservation, both from the point of view of structure and soil quality. High QBS-ar values were obtained in the FIR, BEP, BEV, and ASH forest sites in agreement with many studies conducted on forest soils of various types (Blasi et al., 2013; Galli et al., 2014, 2015, 2021; Menta et al., 2017; Parisi et al., 2005; Szigeti et al., 2022). However, in these soils, the values obtained show great variability that may be due to microhabitat differences in the different study areas, such as cover, slope, and availability of organic matter, but also trampling due to recreational tourism (Blasi et al., 2013; Maharning et al., 2009; Menta et al., 2017). Among different forest coenoses, no significant difference was found in the QBS-ar values recorded, as forests generally tend to have well-structured and stable micro-arthropod communities due to abundant litter and continuous input of organic material (Blasi et al., 2013; Menta, 2012; Menta et al., 2010, 2017). In particular, groups such as Symphyla, Pauropoda, Diplura, Chilopoda, and Diplopoda are typical of stable and undisturbed soils (Bedano et al., 2006; Menta et al., 2008, 2014) and are found in most replicates of these sites, where a high number of euedaphic forms have been found (EMI = 20). In agreement with the results obtained by Blasi et al. (2013), no significant difference was found between managed and unmanaged sites. In fact, it seems that silvicultural management (such as coppicing) does not always have an impact on the QBS-ar index or on the presence of soil invertebrates, but it depends from the intensity and type of practices (Latterini et al., 2023; Setälä et al., 2000; Venanzi et al., 2022). This could be due to the fact that the litter of forest soils maintains a high level of organic material and a favorable microclimate throughout the year, allowing the edaphic mesofauna to have a very short recovery time after disturbances such as tree cutting (Bird et al., 2000). Most of the replicates include euhedaphic groups such as pauropoda, dilplura, and protura, but also other groups related to stable soils such as pseudoscorpions, geophilomorpha, and polyxenida. In some replicates, hemiedaphic and euhedaphic beetles are also present. The presence of these groups in almost all replicates accounts for the high QBS-ar values obtained.

As expected, grasslands obtained highly variable, but on average, lower QBS-ar values than forest sites. In these sites, the number of biological forms varied between 8 and 15. Our results are in agreement with several other studies where grasslands, alpine grasslands, and pasture meadows tend to have lower QBS-ar values than forests and hardly exceed 200 (Gardi et al., 2002; Menta et al., 2008, 2011; Rüdisser et al., 2015). Particularly, in alpine grasslands (like PAS and FdR), the index values vary between 135 and 190 (Leoni, 2008). These habitats seem to have fewer taxa and lower abundance than forest coenoses, probably because the soil is more exposed to climatic stresses compared to forest soils, which are protected by tree canopy and abundant litter (Bird et al., 2004; Callaham et al., 2006; Eaton et al., 2004; Menta et al., 2011). In addition, it should be considered that in some of these environments, the impact of trampling by livestock can be very intense and cause the loss of many taxa, especially in the upper soil layers (Cole et al., 2008; Pietola et al., 2005; Zucca et al., 2010). It is therefore possible that many groups not found at these sites, such as Pauropoda and Diplura, are absent because they make vertical migrations in search of conditions more favorable to their survival (Bedano et al., 2006; Burges & Raw, 1967). Groups such as Pauropoda and Symphyla are less present in the replicates compared to the woodlands and absent in most of them. Diplopoda are present in only one replicate being often associated with forest litter. For the same reason, pseudoscorpions are absent in all replicates. Coleoptera on the contrary are greatly present with various biological forms and in some cases with high EMI values (10 and 15). Despite this, grasslands and pastures generally have high soil fertility and diversity that compensate for lower abundances and contribute to the turnover of organic material in these environments (Tang et al., 2006). Herbaceous formations protect the soil from erosion, and roots allow it to maintain good structure (Gardi et al., 2002; Menta et al., 2011).

Finally, the peatland site obtained the lowest QBS-ar values, but not significantly different from the grassland sites. This may be because some sites such as ALP and PRATO obtained low values comparable to those of the peatland, probably due to differences in disturbance and microclimate in different areas. Most of the euhedaphic microarthropods are absent in the replicates of this site (except for Collembola and Acarina, present in all replicates). Mostly groups with low EMI values such as spiders, Diptera, Hemiptera and Thysanoptera are present. Again, hemiedaphic Coleoptera are present in almost all replicates (EMI 10). This certainly explains the low QBS-ar values obtained. Being the first application, in our knowledge, of the index in this type of environment, it was not possible to have comparative data. In general, peatlands tend to have low diversity and abundance of microarthropods (Silvan et al., 2000), due to the anoxic environment. Despite this low diversity, peatlands are critical for carbon storage, and it is crucial to preserve these environments to mitigate the effects of climate change (Carrera et al., 2011; Humpenöder et al., 2020; IUCN, 2021; Laiho, 2006; Leifeld & Menichetti, 2018; Martini et al., 2006). For this reason, the edaphic fauna that reside there have a role of great importance for the conservation of these ecosystems. It is important to obtain more data on this type of environment, so that we can have more reference values for the QBS-ar index and be able to better monitor the conservation status of these areas.

## Conclusions

The results of this study show that the soils in the Maritime Alps Natural Park of the Marguareis Natural Park are in an excellent state of conservation and have a rich edaphic community. Management practices in examined areas do not seem to have a significant impact on the presence of some groups of microarthropods, indicating that proper management in forest areas does not lead to a deterioration of soil quality. Furthermore, in accordance to recent results (Menta et al., 2017, 2018), this study confirms that QBS-ar values in natural areas can be very different, depending on the environment in which the study is conducted. It is therefore important to establish quality classes for QBS-ar values that differ not only in natural environments, agricultural lands, and urban parks, but also in habitat type.

QBS-ar turns out to be a simple, effective, expeditious, and inexpensive method for assessing soil quality. Therefore, this index is ideal for long-term monitoring and for assessing the impact of certain management practices and disturbances in soils of different types, both natural and agricultural (Blasi et al., 2013; Madej et al., 2011; Maienza et al., 2022; Menta et al., 2011). Moreover, monitoring habitats at risk of desertification such as forests, grasslands, and peatlands through these biotic indices is fundamental.

## Data Availability

The data that support the findings of this study can be accessed by contacting Tommaso Fusco or Andrea Di Giulio upon reasonable request.

## Abbreviations

a.s.l.: Above sea level
CN_LAB: National Centre for the Italian Laboratories Network
COBIODIV: *Conoscenza della Biodiversità*—Biodiversity Knowledge Project of Interreg
EEA: European Environmental Agency
EMI: Eco-morphological index
et al.: *Et alia*
FAI: Abundance-Based Fauna Index
IBQS: Synthetic Index of Biological Quality of Soil
Interreg: Interregional cooperation programme, co-funded by the European Union
ISPRA: Italian Environmental Protection and Research Institute
NBFC: National Biodiversity Future Center
*p*: Probability value
QBS-ar: *Indice di Qualità Biologica del Suolo basato sui microartropodi*—Index of Soil Biological Quality based on microarthropods
QBS-BF: Index of Soil Biological Quality based on microarthropod Biological Forms
QBS-c: Indice di Qualità Biologica del Suolo basata sui Collemboli—Index of Soil Biological Quality based on Collembola
QBS-e: Index of Soil Biological Quality based on earthworms
